# Editorial: The arts therapies and neuroscience

**DOI:** 10.3389/fpsyg.2025.1650846

**Published:** 2025-09-11

**Authors:** Noah Hass-Cohen, Sharon Vaisvaser, Rebecca Bokoch, Christianne E. Strang

**Affiliations:** ^1^Alliant International University, San Diego, CA, United States; ^2^School of Society and the Arts, Ono Academic College Kiryat, Ono, Israel; ^3^The University of Alabama at Birmingham, Birmingham, AL, United States

**Keywords:** creative arts therapies, neuroscience, psychoneurobiology, research, theory, trauma

We are so pleased to welcome you to this Research Topic aimed to advance the emerging knowledge of integrated arts therapies and neuroscience (IATN). It has been an honor to be a part of this collaboration that has showcased a diversity of research methods, art-based modalities, and clinical applications integrating knowledge from neuroscience and the arts therapies. We worked together as an international team to elucidate the unique connections and dialogue between art therapies and psychoneurophysiological functions.

The overarching framework for this Research Topic was captured by an extensive scoping review resource paper by two of our four editors, Bokoch et al. who provided an overview of the current state of IATN publications (*N* = 84) using standardized PRISMA-Scr guidelines. This review surveyed, summarized, and compared arts-based modalities, populations, settings, presenting problems, methods, measures, and outcomes, as well as discussed implications for clinical applications and future research directions. The review found that the most common IATN modality was art therapy, the most common IATN methodology was case study, and the most common research context was medical arts therapies.

Neurobiological mechanisms and underpinnings were the most common topic of the articles submitted to this Research Topic. Each member of this editorial team further contributed to this issue by addressing current knowledge of IATN neurobiological mechanisms, underpinnings and limitations. Hass-Cohen and Clay proposed memory reconsolidation (MR) as a core mechanism in arts therapies, illustrating MR though empirically supported art therapy relational neuroscience drawing protocols. They also showed how IATN interventions can help reprocess traumatic autobiographical distress by meeting neuroscience-informed MR conditions. Vaisvaser discussed selfhood as constructed by predictive processing and interpersonal neural synchronization, situating the arts as a vehicle for neuroplastic reshaping of the multidimensional self. Bridging neuroscience and subjectivity, she highlighted how functional crosstalk between brain networks supports the integration of bodily experiential and narrated mental aspects of the self, their disruptions in psychopathology, and their relevance to the psychotherapeutic use of the arts. Bokoch et al. found that IATN research outcomes suggested improvements in brain activity and functioning, cognitive, affective, sensory, and social functioning, as well as psychological symptoms and behaviors. Changes were captured based on biophysiological measures, neuropsychological assessments, standardized psychological self-report measures, and qualitative reports (Bokoch et al.). Strang identified the current state of evidence underlying concepts in neuroscience that are regularly integrated into IATN theory and practice. She explored the current scope, ethical concerns, and methodological challenges of arts therapies and neuroscience integration and cautioned against uncritical use of mechanistic explanations of arts therapies based on neuroscience explanations. She outlined limits and opportunities for evidence-based integration.

Across the manuscripts included in this Research Topic, our esteemed contributors joined in this endeavor, exploring how arts therapies potentially engage brain networks and physiological systems to promote regulation, physiological arousal, insight, neuroplastic change, and other therapeutic outcomes. Quinn explored how art therapy stimulates the large-scale brain networks (default mode, salience, and central executive networks) in individuals with substance use disorders, suggesting that therapeutic insight and reward are mediated by these systems. Golland et al. connected social play and the noradrenaline system in aging adults, suggesting that arts-based play enhances cognitive flexibility and emotional wellbeing via neurobiological engagement. A case study combining deep brain reorienting and relational art therapy for dissociative identity disorder, exemplifying neurobiologically grounded trauma treatment was provided by Gerge et al. Malhotra et al. also illustrated a neuroscience framework for understanding art therapy's impact on posttraumatic stress disorder (PTSD) through key strategies, specifically metaphors, concretization, and emotional regulation. Shi et al. demonstrated the neurophysiological effects of mindfulness-based music listening in insomnia, with improved mood and attentional processes reflected in behavioral and event related potential (ERP) data. Ding et al. conducted a meta-analysis of rhythm-based interventions for autism, attributing improved social communication in autism potentially through neural synchrony and neuropeptide effects. Related to the social location of these ideas, Kruk-Borisov advocated for anti-racist neuroscience-informed practice, identifying institutional barriers and responsibilities for the field.

Our authors integrated neuroscientific approaches with the following arts modalities. Art therapy, encompassing drawing, crafting, painting, and mask-making, were examined by Arslanbek et al., Hass-Cohen and Clay, and Wolf and Rattigan. Their studies, along with contributions from Gerge et al., Huss et al., Malhotra et al., and Quinn, highlighted the therapeutic potential and transformative power of creativity. Huss et al. compared photo-based and hand-drawn interventions, while Arslanbek et al. focused on the therapeutic benefits of mask-making. Dance and movement therapies, exemplified by Haeyen's study on breathwork and movement as regulatory tools, demonstrated the importance of physical expression in maintaining mental health. Drama and psychodrama were explored in various contexts, including narrative enactments in therapy and intergenerational learning. Colleagues Gerge et al., Peeples et al., and Quinn shed light on how dramatization could facilitate healing and understanding. Music, particularly guided imagery and music (GIM) was another focal area explored by researchers such as Heiderscheit, Ding et al., Shi et al., and Reitere et al.. Their studies revealed the profound effects of musical interventions on emotional and psychological wellbeing. Play and playfulness were embraced by researchers like Golland et al., Haeyen, and Quinn. Their work on playful interventions and play-based reward activation underscored the significance of play in therapeutic settings. Poetry, bibliography, and narrative were explored by researchers such as Peeples et al., Kruk-Borisov, and Wolf and Rattigan. Their studies on narrative learning, bibliographic exposure to anti-racist literature, and narrative interpretations of art highlighted the power of storytelling and reflection in the integrated arts therapies and neuroscience field. Collectively, these studies illustrated the breadth and depth of expressive therapies, showcasing their potential to heal and transform.

A variety of inquiry methods were used. These included: theoretical and conceptual papers by Golland et al., Haeyen, Hass-Cohen and Clay, Malhotra et al., Quinn, Strang, and Vaisvaser, which contributed significantly to the IATN body of knowledge. Scoping and systematic reviews by Bokoch et al., Ding et al., and Reitere et al. provided critical insights and overviews of the field. Qualitative studies using thematic analysis were conducted by Heiderscheit, Kruk-Borisov, Peeples et al., Spee et al., and Wolf and Rattigan. Case studies and vignettes by Gerge et al., Hass-Cohen and Clay, Quinn, Wolf and Rattigan and Strang exemplified the practical applications and implications of IATN.

Clinical innovation for the treatment of mental health conditions, developmental disorders, and medical issues were incorporated across the different publications methods and as listed in alphabetical order included: aging-related cognitive decline (Golland et al.), autism spectrum disorder (Ding et al.), dementia (Peeples et al.), dissociative identity disorder (Gerge et al.), eating disorders (Heiderscheit), insomnia disorder (Shi et al.), neurodevelopmental and neurological disorders (Reitere et al.), Parkinson's disease (Spee et al.), PTSD (Gerge et al.; Hass-Cohen and Clay; Malhotra et al.) substance use disorders (Quinn), as well as traumatic brain injury (TBI) and post-concussive syndrome (Wolf and Rattigan). Adding to ideas about clinical innovation, Reitere et al. reviews how telehealth arts therapies for neurological and neurodevelopmental conditions can support ethical access to support wellbeing.

A nuanced view of the contributions suggested perspectives and practices related to IATN that are trauma-focused, embodied, and centered on\devoted to emotion regulation. IATN and trauma-focused perspectives highlighted how arts therapies address trauma through non-verbal expression, memory reconsolidation, nervous system regulation, and meaning-making. Arslanbek et al. found that viewer responses to trauma-themed masks are shaped by their own histories of adversity, highlighting how trauma resonance affects aesthetic experience. Gerge et al. demonstrated that combining neurobiologically grounded therapy with visual expression addresses dissociation, trauma memories, and embodied fear. IATN and polyvagal theory and techniques were suggested by Haeyen. Hass-Cohen and Clay showed how drawing-based protocols aligned with MR theory, allowing clients to access, destabilize, and update traumatic autobiographical memories. Heiderscheit underscored through thematic analysis of GIM with eating disorder clients, how music therapy supports identification of trauma-related emotional processing, reconnection with the self, and transformation, while mapping narratives onto a hero's journey recovery arc. Malhotra et al. synthesized neuroaesthetic and psychotherapeutic literature to propose a neuroscience-based rationale for using art therapy in PTSD treatment, identifying core mechanisms such as emotion regulation, narrative restructuring, and metaphor. Wolf and Rattigan highlighted caregiver narratives of traumatic brain injury (TBI), using visual imagery to express symptoms that evade verbal or medical recognition, therefore emphasizing the communicative and diagnostic role of IATN in trauma care.

An embodiment and mood regulation theme centered on how somatosensory, visual, and aesthetic experiences regulate emotion and enhance mood through embodied processing. Haeyen described how embodied noticing and naming practices in therapy (e.g., movement, breath, tactile engagement) help regulate stress responses and promote safety. Shi et al. showed that embodied music practices such as mindfulness-based music listening can directly influence affective and attentional states. Huss et al. compared photographed flowers, hand-drawn flowers, and mandalas to showcase how photos evoked stronger embodied emotional shifts, while mandalas offered cognitive stimulation. Publications in this theme examined interpersonal resonance, therapeutic alliance, and group engagement as essential mechanisms in arts therapies. Heiderscheit highlighted how client-therapist relationships and intrapersonal dynamics transformed through GIM. Peeples et al. showed how intergenerational narrative and art-based engagement reduced stigma and increased empathy among psychology students. Vaisvaser linked interpersonal brain synchronization to psychotherapeutic change, fostered by the bodily-anchored relational aesthetic engagement in arts therapy.

In summary, key insights of this special edition illustrated the potential for the validation of IATN mechanisms through neural networks, physiological systems, and neuroplasticity. IATN theories, approaches, and clinical interventions were shown by our authors to provide unique tools to express, regulate, and externalize emotions, as well as reconsolidate trauma memories beyond verbal processing. Furthermore, sensorimotor, visual, and aesthetic elements within the therapy elicited profound emotional shifts and aid in mood regulation. Relational and social processes formed another vital component, with the therapeutic alliance and social play facilitating healing, particularly in cases focused on trauma, stigma reduction, and symptoms related to aging. The empowerment and meaning making fostered by art offer autonomy, insight, and transformation, especially for chronic conditions and marginalized voices. However, the field continues to face several challenges, including the necessity for methodological rigor, diversity, and ethical sensitivity, and innovation. These aspects are crucial for advancing future IATN research and applications.

